# Insights into the optoelectronic behaviour of heteroatom doped diamond-shaped graphene quantum dots

**DOI:** 10.1039/d4ra00603h

**Published:** 2024-04-18

**Authors:** Yassine El Haddad, Hala Ouarrad, Lalla Btissam Drissi

**Affiliations:** a LPHE, Modeling and Simulations, Faculty of Science, Mohammed V University in Rabat Rabat Morocco lalla-btissam.drissi@fsr.ac.ma b.drissi@academiesciences.ma; b CPM – Centre of Physics and Mathematics, Faculty of Science, Mohammed V University in Rabat Rabat Morocco; c College of Physical and Chemical Sciences, Hassan II Academy of Sciences and Technology Rabat Morocco

## Abstract

In this study we aim to manipulate the optoelectronic and photoluminescence properties of diamond-shaped graphene quantum dots (DSGQDs) in order to make them suitable for solar cells and photovoltaic devices. Using DFT and performing many-body effects studies, we investigate the impact of N, B, O, P and S heteroatom doping on DSGQDs in three different positions, namely the zigzag edge, the armchair corner and the surface, in order to identify the most appropriate and promising configurations. All the doped GQDs are found to be chemically stable making it possible to realize them experimentally. Additionally, the obtained results show that substitution with heteroatoms has a remarkable effect on the electronic energy gap, noticeably decreasing it. Doping also has a significant effect on the optical response by shifting the absorption peaks towards the visible energy range. The excitonic behaviour has revealed that these nanostructures are potential candidates for photovoltaic devices. One can deduce that doping DSGQDs with heteroatoms is useful and promising for the targeted applications.

## Abstract 2

Investigation of heteroatom (N, B, O, P and S) doping impact on the structural, optoelectronic and photoluminescence properties of diamond-shaped graphene quantum dots for photovoltaics and nanomedical applications, and energy technologies.

## Graphical abstract

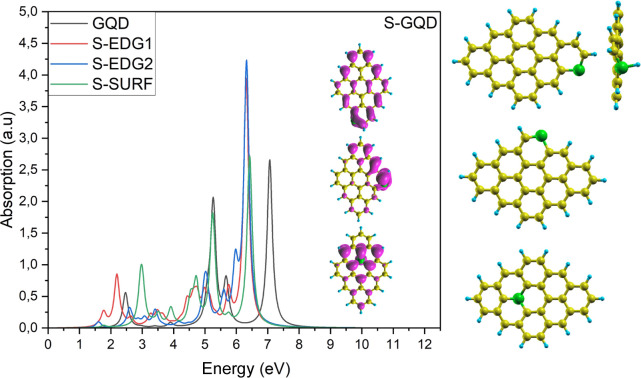

## I. Introduction

Since the first exfoliation of a 2D graphene monolayer in 2004,^1^ this hexagonal lattice of carbon atoms has received a lot of attention from scientists for its excellent properties,^2^ in particular, the charge carriers of graphene that can reach very high mobility values,^3,4^ the high thermal and chemical stability,^5^ the excellent optical transparency,^6^ its absorption in a very wide energy range,^7^ the low resistivity and the high mechanical strength.^8^ Graphene is interesting for photonics,^9^ microelectronics, in particular for the production of transistors,^10^ and so on. However, it is a zero gap semi-conductor,^11^ which reduces its application in optoelectronics.^12^

Many promising strategies, have been introduced to create a band gap in graphene-based materials, including the addition of a transverse electric field, surface chemical functionalization and the hybridization between atoms.^13–15^ Another strategy consists of quantum confinement of electrons along the armchair and zigzag edges by producing 1D graphene-nanoribbons^16^ or 0D graphene quantum dots (GQDs) which is the subject of the present work. To manufacture GQDs, two main approaches have been adopted:^17–20^ namely the top-down method which relies on cutting macroscale systems, such as graphite, carbon black or carbon fibers, to obtain nanoscale quantum dots; and the bottom-up approach based on organic chemistry, which aims to generate GQDs from small-scale systems to a larger one. GQDs are therefore small fragments of graphene having excellent and tunable features, namely gap energy, optical absorption, photoluminescence and quantum confinement effects.^21,22^ These excellent properties make these zero-dimensional nanostructures more attractive for optical and optoelectrical devices used in industrial and medical fields, such as, photovoltaic devices, catalysis (electrocatalysis, photocatalysis) bio-imaging, medical diagnosis.^23^ Several experimental and theoretical studies have shown that it is possible to control and enhance the optical and electronic properties of GQDs through the adjustment of size,^24^ edge configuration,^25^ shape,^26^ chemical functionalities,^27^ heteroatom doping^28^ and defects^29^ for desired and specific applications.

Interestingly, heteroatoms doping of 5 to 10 nm diameter GQDs, like nitrogen (N), has been successfully done using high-yield hydrothermal methods.^30^ The resulting circular structures (N-GQDs) emit a strong blue emission under 365 nm UV light excitation and exhibit a high fluorescence quantum yield up to 75.2%, which is close to the conventional semiconductor QDs. Consequently, these high-yield N-GQDs seem to be promising for new avenues in the field of cell labelling, bioimaging and environmental detection.^30^ In the same context, a hydrothermal approach has been developed for the synthesis of N-GQDs of about (1–7 nm) in diameter and a ratio atomic N/C of 5.6%, which are characterized by bright blue photoluminescence and excellent up-conversion luminescence properties.^31^ Otherwise, the theoretical study^32^ on N-doping circular graphene quantum dots with a diameter ranging from 1 to 5 nm, reveals that nitrogen edge doping improves the fluorescence properties of GQDs compared to non-doped structure, and causes a blue or red shift in the absorption and emission spectra depending on the hybridization of the boundary orbitals. It was also shown that the effect of N-doping is related to the different N-doping pattern and positions, namely pyrazole, pyridazine, graphitic, pyridinic at center or edge, pyrrolic at center or edge, amido at center or edge.

Other types of substituent have also been investigated. Sulfur doped GQDs (S-GQDs), with a spherical shape and an average diameter of 2.46 nm, have been synthesized by the co-combustion method (TXJ method) of a liquid mixture of paraffin oil and sulfide of carbon (CS_2_) as a source of carbon and sulfur. These nanostructures show a blue shift of the absorption peaks compared to the non-doped GQDs, which indicates an increment in the absorption of the S-GQDs in the ultraviolet-visible region.^33^ Besides, round-shaped sulfur-doped GQDs with a relatively uniform size of about 5.2 nm have been prepared *via* a hydrothermal process using fructose and sulfuric acid as the raw materials. They can emit a variety of colors, covering most colors in the visible light region, compared to pristine GQDs which have only two absorption peaks centred at 228 and 282 nm.^34^ Similarly, S-GQDs with a narrow size distribution of about 3 nm and with phenolic hydroxyl decorated edges, synthesized by the top-down method from the graphite quantum dots based on sulfur-doped graphene, have shown blue-green fluorescence and improved electronic properties compared to non-doped GQDs, which renders S-GQDs potential candidates for a fluorescent probe for the detection of Fe^3+^ ions.^35^

Phosphorus-doped graphene quantum dots (P-GQD), characterized by their excellent monodispersity, a spherical shape and a small and narrow size distribution mostly in the range 2 to 4 nm, have been synthesized *via* a bottom-up electrochemical approach with a high P/C ratio; showing excellent ability to scavenge free radicals and several potential uses in biotechnology and medicine.^36^ Likewise, P-GQDs have also been prepared with different concentrations of emission wavelengths (457–632 nm) and high quantum efficiency (0.54–0.73) for application in bioimaging.^37^ Furthermore, oxygen doping is typically studied using specifically engineered GQDs that inherently contain oxygen-rich chemical groups capable of altering the electronic characteristics of the GQDs. Indeed, O-rich groups make an important contribution in observing blue shifted absorption peaks in the photoluminescence emission of experimentally synthesized N-GQDs due to the localization of electron–hole pairs, which gives GQDs excellent hydrophilicity, unique optical performance, favourable biocompatibility and no toxicity.^38^ Furthermore, an electrochemical method was adapted to synthesize functional GQDs characterized with a uniform size of 3–5 nm and exhibiting a green luminescence.^39^ These structures have O-containing groups on the surface which makes them soluble in aqueous media and suitable for functionalization and various applications such as lasing, and light emitting diodes.

Inspired by all of these experimental works exploring the effect of heteroatoms on the optoelectronic properties of GQDs, we aim to examine how heteroatoms impact the physical properties of diamond-shaped graphene quantum dots (DSGQDs), which we have previously investigated and found to exhibit strong stability alongside intriguing structural and electronic characteristics. Furthermore, DSGQDs display significant absorption and photoluminescence properties in both the visible and infrared regions of the solar spectrum, making them highly relevant for various applications. In this study, our objectives are twofold: (i) investigate the impact of heteroatom doping—employing N, B, O, P, and S heteroatoms—on the optoelectronic properties of DSGQDs. These DSGQDs possess a distinctive diamond shape characterized by zigzag edges and armchair corners connected by a seam atom. Our focus is to uncover the fundamental alterations induced by heteroatom doping in these properties. (ii) Explore the potential utilization of heteroatom-doped DSGQDs across various fields, including optoelectronics and photovoltaics. Specifically, we aim to explore their applicability in devices such as light-emitting diodes (LEDs) and solar cells. Additionally, we aim to investigate their potential in nanomedical applications, particularly in diagnostic devices and drug delivery systems, as well as their suitability for use in energy storage and conversion technologies. Based on our previous publications,^51–55^ the shape of DSGQDs was found to have a considerable effect on the optoelectronic, excitonic and photoluminescence behaviours, especially upon functionalization. So exploiting these findings, we focus on studying the special response of DS nanostructures to the heteroatom doping process and its associated factors as a function of the type and position of five dopants, including zigzag or armchair corners, or inside the surface. Notice that the choice of these five heteroatoms is mainly for their nanomedical and safety use as proven in experimental works cited in previous sections. To do so, three different doping configurations are modelled and investigated for each heteroatom. Our findings have shown, among others, that the heteroatom doping has noticeably affected the electronic energy gap as well as the absorption character of pristine GQDs through shifting the absorption peaks towards smaller energy ranges. All these findings elect the heteroatom doping process as a useful strategy to adjust the GQDs behaviour and to tune the absorption and the photoluminescence of GQDs, making them promising for photovoltaic devices and nanomedical applications.

This paper is organised as follows, we start by detailing the computational method used for performing our calculations, then we report the findings and discuss the obtained results in the next section. We summarize with a conclusion.

## II. Computational details

We aim in this work, to determine the electronic and optical properties of our graphene-based quantum dot structures. The numerical simulation procedures that we used are the following. First, we performed geometry optimization and ground state calculation using density functional theory (DFT) with a plane wave approach in the generalized gradient approximation (GGA) with a Perdew–Burke–Ernzerhof (PBE) exchange-correlation function,^41^ implemented in the quantum espresso (QE) simulation package.^42^ We used conserved norm pseudo-potentials and a set of plane wave bases with a kinetic energy cutoff of 60 Ry. In order to avoid non-physical interactions between periodic images in the study of isolated systems, a 20 Å vacuum is used in all three dimensions. To build the dielectric matrix and Green's function, we used a total of 600 bands for all structures. In all the calculations, a single gamma point is taken into account due to the zero-dimensional character of the structures considered. The considered configurations are totally optimized under stresses and forces until the components of all the forces are lower than 10^−3^ eV.

In the second step of simulation, we ensure precise quasiparticle energies by utilizing the many-body perturbation theory (MBPT) within the GW approximation. This method relies on the Green's function (*G*) and the projected Coulomb interaction (*W*).^41^ Our calculations are carried out using the Yambo code.^44^ The quasiparticle (QP) corrections to the GGA eigenvalues are determined using the followed equation:

1 *E*^QP^_*nk*_ = *E*^QP^_KS_ + 〈*Ψ*_*nk*_|∑(*E*^QP^_*nk*_) − *V*^KS^_*xc*_|*Ψ*_*nk*_〉

where 〈*Ψ*_*nk*_| is the wave-function, *E*^QP^_KS_ the Kohn–Sham (KS) energy, *V*^KS^_*xc*_ the exchange-correlation potential and ∑(*E*^QP^_*nk*_) is the self-energy operator.

Moreover, we studied the optical behaviour of the structures by solving the Bethe–Salpeter equation (BSE) combined with the GW method. It is generally rated GW + ESB.

We studied the excitonic properties using the Bethe–Salpeter (BSE) equation:2
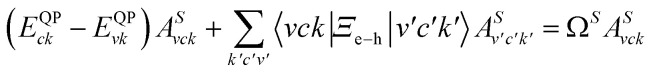
where *Ξ*_e–h_ is the electron–hole interaction kernel, Ω^*S*^ the excitation energy and *A*^*S*^_*vck*_ is the exciton amplitudes.

Charge-transfer is calculated using Bader analysis.^43^

## III. Results and discussion

In this work, we study the effect of heteroatom dopants on the electronic and optical properties of graphene-based quantum dots (C_30_H_14_). For this, the five dopants X = B, N, O, S, and P, are used since they are the most commonly used in literature for the intended applications mentioned above.

### A. Structural properties

We focus on a graphene-based quantum dot (C_30_H_14_) containing a total of 44 atoms of which 30 are carbon atoms forming the core while 14 are hydrogen atoms passivating the QD edges; the bonds hanging from the edge are attached to hydrogen atoms in order to guarantee the good stability of our structure.^51,52,58,59^ Successfully fabricated, these QDs, referred to as dibenzo[*bc*,*kl*]coronene, combine between zigzag edges and armchair corners,^45–48^ and the carbon atoms in the ring structure are sp^2^ hybridized leading to a planar geometry.^51^ The GQD-C_30_H_14_ structure is considered as the most appropriate configuration to be doped among its counterparts discussed in our earlier works, notably pyrene with 16 carbon atoms (C_16_H_10_) and C_48_H_18_ QDs do not yet have a suitable synthesis route.^52,53^ Furthermore, dibenzo coronene exhibits the most suitable features for our aimed applications.^54,55,57^

**Fig. 1:**
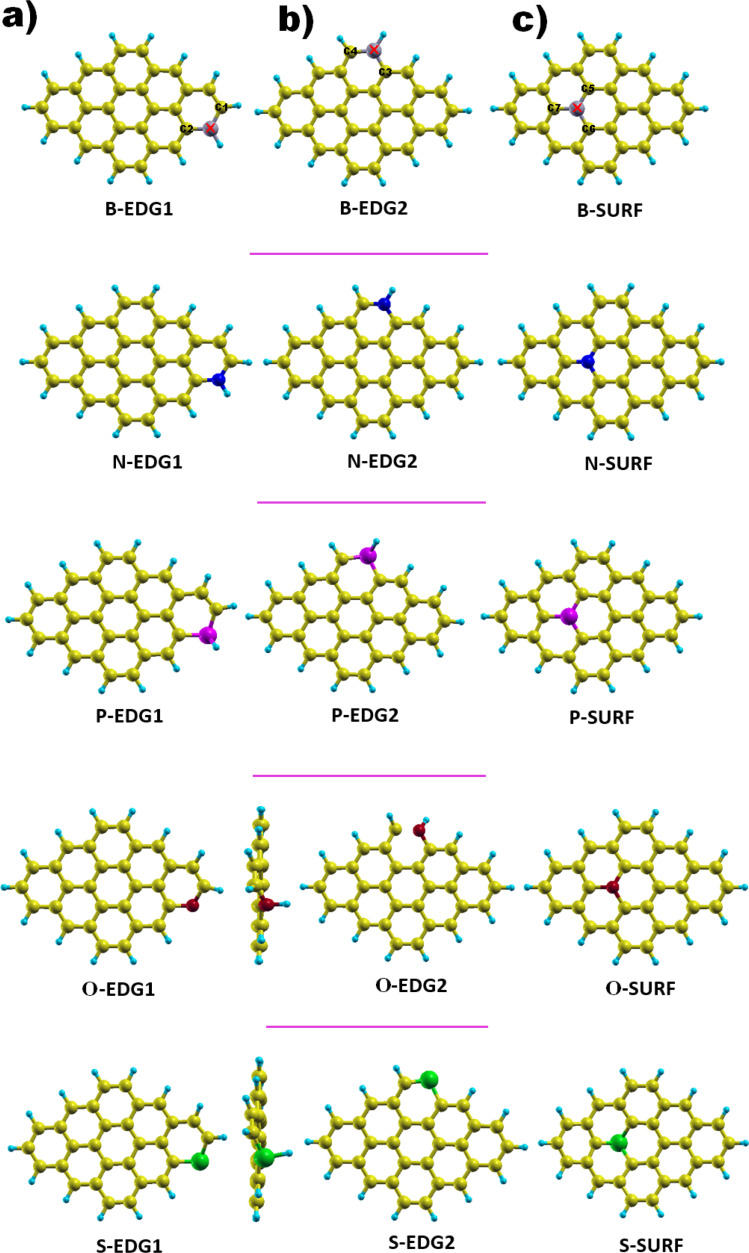
GQD structures doped with X = B, N, P, O and S heteroatoms in three positions. (a) X-EDG1 position, (b) X-EDG2 position and (c) X-SURF position.

As illustrated in Fig. 1, each dopant X is placed in three possible positions: (i) the zigzag edge (X-ZZ) we refer to as X-EDG1, (ii) the armchair edge (X-AC) denoted also as X-EDG2 and (iii) inside the surface (X-IN) or (X-SURF).

The covalent bond lengths and the angles between the dopants and their adjacent carbon atoms, listed in Table 1, reveal that doping leads to structural distortion of the GQDs. Indeed, starting with the inside surface position (X-IN), we notice that the C–C bond with a bond length of about 1.4 Å in pure GQD increases upon doping to 1.54 Å, 1.49 Å, 1.65 Å and 1.77 Å for *d*_OC_, *d*_BC_, *d*_PC_ and *d*_SC_, respectively. Similar enhancement of the X–C bond length is also observed for the zigzag-edge position as well as the armchair-edge position. However, we should note that the nitrogen atom keeps the value of the covalent bond (1.41 Å) close to that of the pristine structure because the atomic radius of the C and N atoms are almost equal. One can also notice that when comparing the interatomic distances for the same dopant, like *d*_*XC*_3__ with *d*_*XC*_4__, the increase is more pronounced in the AC-configuration with respect to the ZZ-edge case, especially for P, O and S, while it is less significant for B and N. Therefore, we conclude that the distortion in our structures is originally induced by the length modification of the X–C bond brought by a doping process which mainly depends on the variation of the atomic radius size and the nature of the neighbour atoms.

**Table 1:** The interatomic distances and the angles for heteroatom doping of C_30_H_14_ in ZZ, AC and IN configurations

X	Bond length (Å)							Angles (°)			
	ZZ		AC		IN			ZZ	AC	IN	
	*d*_*XC*_1__	*d*_*XC*_2__	*d*_*XC*_3__	*d*_*XC*_4__	*d*_*XC*_5__	*d*_*XC*_6__	*d*_*XC*_7__	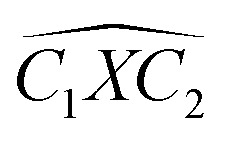	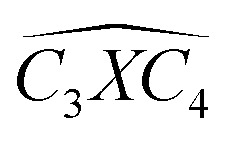	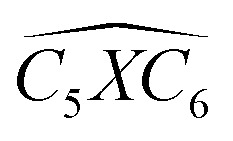	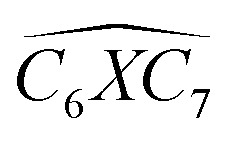
B	1.53	1.54	1.54	1.50	1.49	1.49	1.48	116.5	115.7	120.1	119.8
N	1.38	1.37	1.39	1.37	1.41	1.41	1.41	122.4	124.1	119.5	120.2
P	1.79	1.82	1.81	1.75	1.65	1.65	1.65	100.6	102	119.7	120.1
O	1.50	1.51	1.44	1.71	1.53	1.53	1.54	113.2	114.9	115	114.1
S	1.73	1.75	1.67	1.78	1.76	1.77	1.77	103.1	102.4	100.6	100.7

Furthermore, the bond angles of 
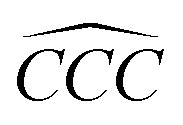
 remain at almost 120° in all structures, like the pure case, whereas the situation is different for 
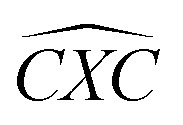
 angles. Indeed, in the IN-configurations the angle 
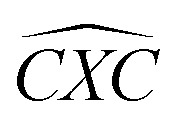
 is still close to 120° for B, N and P, a signature of the hexagonal lattice with sp^2^-hybridization, however it becomes much lower for the O and S-adsorbent. This latter effect is also observed when the X-atom is located at the zigzag edge or at the armchair one, which indicates that the doping induces a sort of buckling around the substitution region where the sp^2^-hybridization is significantly affected, as shown in Table 1, and the obtained angle values vary between 100° and 103°.

In summary, the P, S and O atoms cause significant structural modifications upon doping the pristine structure. However, the N and B atoms cause less distortion. On the other hand, it should be specified that the armchair O-doping leads to cracking of the covalent bond between the oxygen atom and the adjacent carbon atom, which can affect the electronic and optical properties of this structure.

### B. Energetic stability

In order to evaluate the energetic stability of the studied structures, we calculated the cohesive energy values *E*_coh_ of the doped structures. The cohesive energy *E*_coh_ values are calculated as follows:^60^3
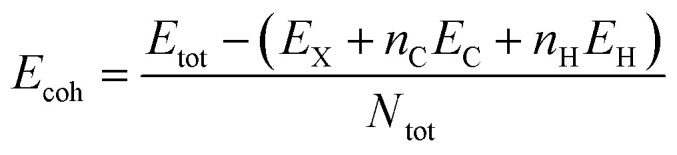
where *E*_tot_ the total energy of doped GQD. *E*_X_ is the energy of the substituent atom X = B, N, O, P and S, *E*_C_ is the energy of the C atom, and *E*_H_ is the energy of the H atom. *n*_C_ and *n*_H_ are the number of C and H. And finally, *N*_tot_ is the total number of atoms.

Table 2 lists the values of the cohesive energy *E*_coh_ obtained *via* DFT calculations. More negative values confirm the greater stability of the investigated structures.^60^ Indeed, all calculated cohesive energy values *E*_coh_ are negative, confirming the stability of all the studied systems is in good agreement the previous report.^60^ The pristine GQD structure has a value of 7.270 eV which decreases after doping with boron, nitrogen, oxygen, phosphorus or sulfur. This change in the cohesive energy value can be explained in terms of structural properties. The sulfur and phosphorus doped systems show the greatest decrease in cohesive energies, followed by the oxygen doped structure, as shown in Table 2. P and S doping results in structural distortions of the hexagonal ring by increasing the bond length from 1.42 to 1.8 Å, which decreases the values of the bond energies. In contrast, N and B doping results have shown a slight drop in cohesive energies compared to the pristine system, which can be attributed to the almost similar ionic radii between the B, N, and C atoms, with little change in the length of the C–N and C–B bonds.

**Table 2:** The cohesive energy values *E*_coh_ of the studied systems, in eV

Structures	GQD	B-ZZ	B-AC	B-IN	N-ZZ	N-AC	N-IN	P-ZZ	P-AC	P-IN	O-ZZ	O-AC	O-IN	S-ZZ	S-AC	S-IN
*E*_coh_	−7.270	−7.198	−7.189	−7.181	−7.244	−7.234	−7.221	−7.158	−7.144	−7.106	−7.131	−7.131	−7.098	−7.107	−7.106	−7.076

### C. Stability indices

In order to gain insight into the chemical stability and chemical reactivity of the doped GQDs, we calculate the stability indices, namely the electrophilicity (*ω*) expressed as follows:^49^4
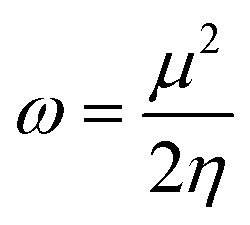


and the hardness (*η*) and the chemical potential (*μ*) given in terms of the calculated frontier molecular orbital energies; *E*_HOMO_ and *E*_LUMO_:^49^5
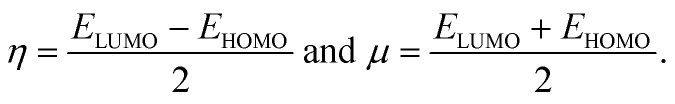


As shown in Table 3, heteroatom doping brought considerable modifications to the chemical stability of the pristine GQDs. Starting with the hardness, it is noticeable that the doping mechanism decreases the hardness of the investigated structures making them slightly soft and thus more polarizable and easy to be excited. This result can be afforded to the distortion brought by the heteroatom doping in good accordance with ref. 61, where the hardness has been decreased upon doping the asphaltene structure. Whereas, for the electrophilicity, we can clearly observe that the edge-doping position reduces the electrophilicity while the IN-doping position increases it which concords well with ref. 62, showing how the doping mechanism affects the chemical potential and electrophilicity index of h-BN QDs differently depending on the doping position. Our findings can be attributed to the difference in charge transfer related to the nature of the neighbor atoms and thus, the significant frontier orbital hybridization.

**Table 3:** Global reactivity descriptors: hardness *η*, chemical potential *μ* and electrophilicity *ω*, as well as gap energy *E*^GGA^_g_ and *E*^GW^_g_ at DFT and GW level, respectively, first bright exciton binding energy *E*^X^_b_, optical gap *E*^X^_opt_, and the singlet–triplet energy splitting *Δ*^X^_S–T_. All the energies are in eV

Structures	*η*	*μ*	*ω*	*E* ^GGA^_g_	*E* ^GW^_g_	*E* ^X^_b_	*E* ^X^_opt_	*Δ* ^X^_S–T_
GQD	0.6585	3.4754	9.171	1.32	4.28	1.81	2.4	1.62
B-ZZ	0.696	3.063	6.738	1.30	3.326	2.094	1.7217	0.81
B-AC	0.546	3.359	10.324	1.093	2.980	2.179	1.5732	0.76
B-IN	0.632	3.3947	9.105	1.265	3.210	2.070	1.6917	0.97
N-ZZ	0.560	2.3029	4.734	1.120	4.040	2.709	1.3313	0.24
N-AC	0.315	2.568	10.458	0.630	3.435	2.164	1.2713	1.001
N-IN	0.099	2.566	33.256	0.198	3.114	1.443	1.6717	0.59
P-ZZ	0.667	2.649	5.258	1.314	4.253	2.639	1.614	0.32
P-AC	0.354	2.925	12.084	0.708	3.566	2.442	1.1241	0.64
P-IN	0.2656	2.563	12.364	0.531	3.416	2.636	0.7808	0.55
O-ZZ	0.526	2.577	6.314	1.052	3.805	2.363	1.4414	0.91
O-AC	0.460	3.014	9.867	0.921	1.286	0.940	0.3456	0.19
O-IN	0.269	2.982	16.491	0.539	2.236	1.445	0.7908	0.37
S-ZZ	0.522	2.823	7.635	1.044	3.866	2.094	1.7710	1.17
S-AC	0.555	3.145	8.898	1.111	4.193	2.585	1.6088	0.59
S-IN	0.474	3.164	10.548	0.949	3.851	2.079	1.7718	0.74

### D. HOMO–LUMO gap analysis

The HOMO–LUMO (H–L) energy gap obtained using the GGA-DFT and GW methods are listed in Table 3.

The GGA-DFT data reveal that O-, B-, N-, P- and S-dopants either on the edges or inside the surface, significantly affect the band gap of the pristine GQDs in good agreement with our previous work demonstrating how the energy gap is sensitive to substituents^51^ and the functionalization of diamond-shaped quantum dots (DSQDs).^53,54^ More precisely, with respect to the pure nanostructure, dopants result in a reduction of the corresponding H–L gap for the ZZ-, AC- and IN-configurations, in good accordance with ref. 40, showing that N and S impurities remarkably reduce the energy gap of the hexagonal-shaped graphene quantum dots. Furthermore, the IN-doping exhibits the most pronounced decrease in the H–L energy gap of the pure nanostructure for most heteroatoms, except dopants causing significant structural distortion, which disrupts the carbon sp^2^ hybridization in the skeleton due to the higher electronegativity or larger size of impurities, and results in a pronounced reduction of the energy gap as explained for the case of nitrogen doping in carbon dots.^63^ Consequently, the variation of the HOMO or LUMO energy gap is attributed to the hybridization of the frontier molecular orbitals as well as the geometry deformation which mainly depends on the type and the position of heteroatoms, in accordance with the results on doping heteroatoms and covalent bonding with specific groups in hexagonal GQDs.^64^

On the other hand, a significant enhancement brought by the quasiparticle corrections is observed in the GW-energy gap with respect to the GGA-results. This behaviour is attributed to the heteroatoms acting like an electron donor or acceptor which increases the screening and impacts the electron–electron interactions.^53,54^ Our finding are in good agreement with the many-body study of functionalized hexagonal GQDs.^56^

**Fig. 2:**
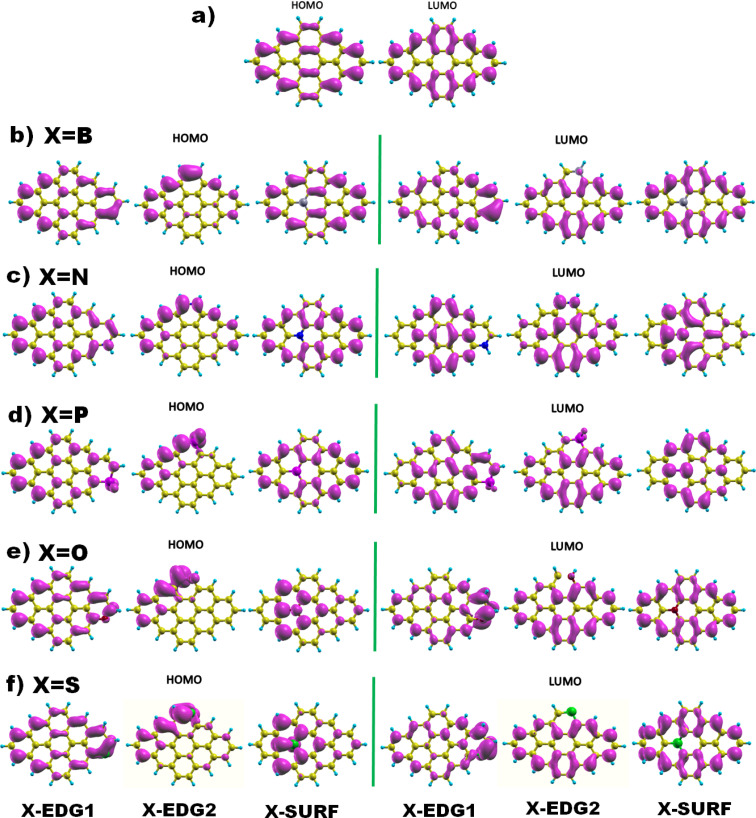
The HOMO and LUMO electronic charge density distribution of (a) pristine, and (b–f) doped DSGQDs with X = B, N, P, O or S.

Next, Fig. 2 displays the charge density distributions associated with the HOMO and LUMO energy levels obtained using the GGA method. Interestingly, most of the investigated structures exhibit an edge-localized charge density similar to the pristine structure. However, there is a wide difference in the atomic contribution depending on the dopant type and its position. Starting with analysing the zigzag-doping case, we can see that for all the structures, a considerable contribution to either the HOMO or the LUMO levels originates from the dopant X. We should note that, for N, P, and O dopants, the contribution to the HOMO originates from carbon atoms in subsite A of the hexagonal structure, while the contribution to the LUMO comes from the the neighboring carbon atoms. This explains the reduction in both HOMO and LUMO energies and thus the decrease in the H–L energy gap values. Whereas GQDs doped with the B atom, which is a donor atom, and which provides a significant contribution from the dopant to both HOMO and LUMO levels, increases the corresponding energies as listed in Table 3, explaining the slight rise in H–L energy gap value with respect to the pristine structure. The same behaviour has been noticed for the S doped structure, even though only the HOMO energy is increased which explains the reduction of the energy gap similar to the case of N, P and O atoms. On the other hand, armchair-edge doping has shown a different impact on the charge density of the pristine structure, depending on the dopant type and the position. We notice that only a few carbon atoms contribute to the HOMO level which increased its energy, while the LUMO energy has shown a small change compared with the HOMO energy, resulting in decreasing the GGA(H–L) energy gap.

In the case of inside the surface (IN or INace) doping, the distribution of charge density for a B doped structure is quite similar to that of the pristine structure, which results in small variation in the H–L gap originating from the absence of the armchair corner contribution. For the N and P doped structure, the contribution to the LUMO level, coming mostly from the dopant and the INace atoms, widely alters the *E*_LUMO_ resulting in a slight increment in the LUMO energy. The HOMO energy undergoes a significant increment since the contribution to the HOMO originates mainly from the zigzag edges characterized with edge states, which results in a quick reduction of the H–L(GGA) gap. The opposite is true for O and S doping, the contribution to the HOMO comes from the dopant and the surface atoms, while the charge density of the LUMO is mostly localized at the zigzag edges, showing a slight decrement in *E*_LUMO_ and an increment in *E*_HOMO_, which explains the diminution in the H–L(GGA) energy gap. To sum up these findings, we can deduce that heteroatom doping, gathered by varying the type and the position of the dopant, considerably alters the electronic properties of the pristine DSGQD and this originates mainly from the competition between the strong frontier molecular orbitals hybridization and also the distortion effects.

### E. Charge transfer and magnetic behaviour

#### 1. Charge transfer

Table 4 shows the charge transfer between different atoms and their neighboring carbon atoms, calculated using Bader analysis.

**Table 4:** Bader charge transfer of dopant X = B, N, P, O or S, (+ve) denotes electron loss while (−ve) denotes electron gain

Structures	B-ZZ	B-AC	B-IN	N-ZZ	N-AC	N-IN	P-ZZ	P-AC	P-IN	O-ZZ	O-AC	O-IN	S-ZZ	S-AC	S-IN
X	+2.99	+2.99	+2.99	−0.35	−0.41	−0.81	+3.40	+3.56	+4.99	−1.42	−1.55	−0.83	+0.36	+0.46	+1.94

We observe that B, P, and S donate electrons to nearby carbon atoms, while N and O atoms take electrons from their neighboring carbon atoms. This behaviour is mainly attributed to the Pauling electronegativities which are highest for oxygen O(3.44), then nitrogen N(3.04), followed by carbon C(2.55), sulfur S(2.58), boron B(2.04), and phosphorus P(2.19). Besides, the atoms shared between heteroatoms and carbon atoms suggest that B, N, and O atoms form polar covalent bonds with carbon neighbors, whereas P and S atoms create nonpolar covalent bonds with carbon atoms.

#### 2. Magnetic behaviour

In this paragraph, we report the results obtained upon performing spin-polarization employing DFT calculations, in order to examine the effect of heteroatom doping on the non-magnetic pristine GQD. Interestingly, and depending on the calculated magnetic moments, we can divide our doped systems into two categories: non-magnetic doped GQDs and magnetic doped GQDs. Specifically, O and S doped GQDs remain non-magnetic, however B, N and P atoms induce the non-magnetic phase of GQD into magnetic nanomaterials. This result is mainly explained by the introduction of localized states within the electronic structure of the GQDs. These localized states can lead to the formation of unpaired electrons, resulting in a net magnetic moment and conferring magnetic properties to the pristine GQDs, paving the way for utilizing these magnetic nanomaterials in spintronic applications.

### F. Absorption profile and excitonic effects

**Fig. 3:**
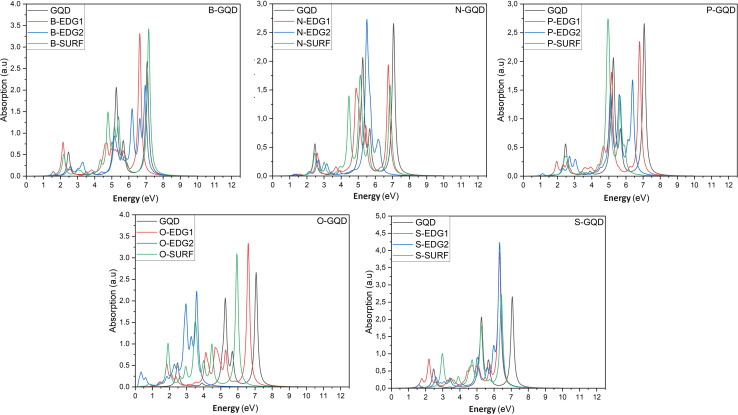
The optical absorption spectra of pristine and heteroatom doping structures with X = B, N, O, P or S, at the GW + BSE level.

In what follows, we investigate the impact of heteroatom doping on the optical profile of DSGQDs through the determination of the absorption spectra in the absence and the presence of the electron–hole interactions. The inclusion of excitonic effects has drastically modified the peak position and the intensity of the first bright exciton making GW + RPA results negligible. For this reason, Fig. 3 only plots the optical absorption spectra at the GW + BSE level for the *X*-direction light polarization. The optical response for light polarized along the *Y*-direction have shown anisotropic behaviour upon doping. Compared to the optical behaviour of the pristine GQD, Fig. 3 shows that the ZZ-, AC-, IN-dopants shift the absorption curves towards lower energies. Interestingly, the O-AC and O-IN structures show the greatest influence on absorption spectra compared to other structures, their absorption curves are the most redshifted. On the other hand, the absorption spectrum of the B-IN-GQD structure is the least affected compared to the other doped structures, followed by the B-ZZ-GQD structure and then the P-ZZ-GQD structure. We should note that the doping process helps enhance the intensity peaks compared to the pristine structure, especially when doping with O, B and S heteroatoms. As a direct consequence of the absorption redshift caused by dopants, the first exciton is manifested by the presence of an absorption peak located at an energy lower than that of the pristine structure. Table 3 shows how the difference in type and position of the dopant allows a series of GQDs with different absorptions in the visible and infrared region of the solar spectrum. Indeed, the majority of the exciton binding energy *E*^X^_b_ values range in the interval [2.07,2.7] eV which prove that our structures are promising candidates for photovoltaic applications. The structures, exhibiting significant GW-gap and thus an optical gap in the visible–infrared region of the solar spectrum, may be potential candidates for different applications, especially for bioimaging.

To predict the type of photophysical process and the effect of the doping mechanism on the luminescent behaviour of our doped GQDs, Table 3 reports the singlet–triplet energy splitting *Δ*_S–T_. Compared to pristine GQDs, the heteroatoms B, N, P, O and S lower the singlet–triplet energy splitting values compared to the non-doped GQD nanostructure. Indeed, the calculated values vary from 1.46 eV for B-IN, down to the lowest value of 0.19 eV obtained for the O-AC configuration. Furthermore, thermally activated delayed fluorescence (TADF) characterizes N-ZZ and O-AC structures, exhibiting the lowest splitting values (less than 0.37 eV).^50^ It follows that fluorescence occurs in QDs with splitting energy values between 0.5 and 1 eV, except for the P-ZZ structure characterized by triplet–triplet annihilation processes, whereas S-ZZ, B-ZZ and B-IN structures exhibit a singlet fission process in agreement with the findings in ref. 50, regarding the control of singlet–triplet splitting for organic exciton manipulation. Our results are similar to other GQDs structures of the same size characterized by TADF, such as molecular structures of SiCQDs edge-functionalized with H, OH and COOH, and edge-functionalized SiQD structures with H and OH.^51,52,54,55^

## IV. Conclusion

In summary, we have adapted a heteroatom-doping mechanism to manipulate the structural, electronic, optical, excitonic and photoluminescence properties of DSQDs. The obtained results show that the type and the position of the dopant, influences the binding environment of the GQD with respect to the pristine nanostructures. Interestingly, the INace doping improved the responsiveness of DSGQDs, especially upon using N, P and O dopants, making them more responsive and less hard. The optoelectronic behaviour of the pristine structure is considerably decreased with doping, depending on the nature and the location of the heteroatom dopant due to the frontier orbitals hybridization. With respect to pure QD, the absorption curves are shifted towards lower energies and the binding energy of the first exciton is significantly increased for most edge-doped structures, except for the B heteroatom which exhibits anomalies. Whereas, for surface-doped structures, the binding energy shows an increase only for the S-dopant. Finally, the singlet–triplet energy splitting, which strongly depends on the doping atom type and site, greatly reduces the intersystem conversion from the singlet state to the triplet state and consequently enhances the fluorescence process. These results seem to be very useful and promising for applications in photovoltaic and optoelectronic devices.

## Data availability

This work has no associated data.

## Author contributions

Yassine Haddad: conceptualization, investigation, methodology, visualization and writing. Dr Hala Ouarrad: conceptualization, investigation, methodology, visualization, writing. Prof Lalla Btissam Drissi: conceptualization, investigation, methodology, visualization, supervision, writing, review and editing.

## Conflicts of interest

The authors declare that they have no known competing financial interests or personal relationships that could have appeared to influence the work reported in this paper.

## Supplementary Material

## References

[cit1] Novoselov K. S., Geim A. K., Morozov S. V., Jiang D., Zhang Y., Dubonos S. V., Grigorieva I. V., Firsov A. A. (2004). Electric Field Effect in Atomically Thin Carbon Films. Science.

[cit2] Biró L. P., Nemes-Incze P., Lambin P. (2012). Graphene: nanoscale processing and recent applications. Nanoscale.

[cit3] Bao W. S., Liu S. Y., Lei X. L., Wang C. M. (2009). Nonlinear dc transport in graphene. J. Phys.: Condens. Matter.

[cit4] Shishir R. S., Ferry D. K. (2009). Intrinsic mobility in graphene. J. Phys.: Condens. Matter.

[cit5] Galashev A. E. E., Rakhmanova O. R. (2014). Mechanical and thermal stability of graphene and graphene-based materials. Physics.

[cit6] Nair R. R., Blake P., Grigorenko A. N., Novoselov K. S., Booth T. J., Stauber T., Geim A. K. (2008). Fine structure constant defines visual transparency of graphene. Science.

[cit7] Bao Q., Loh K. P. (2012). Graphene photonics, plasmonics, and broadband optoelectronic devices. ACS Nano.

[cit8] Lee C., Wei X., Kysar J. W., Hone J. (2008). Measurement of the elastic properties and intrinsic strength of monolayer graphene. Science.

[cit9] Bonaccorso F., Sun Z., Hasan T., Ferrari A. C. (2010). Graphene photonics and optoelectronics. Nat. Photonics.

[cit10] Schwierz F. (2010). Graphene transistors. Nat. Nanotechnol..

[cit11] Gui G., Li J., Zhong J. (2008). Band structure engineering of graphene by strain: first-principles calculations. Phys. Rev. B: Condens. Matter Mater. Phys..

[cit12] Kusmartsev F. V., Wu W. M., Pierpoint M. P., Yung K. C. (2015). Application of graphene within optoelectronic devices and transistors. ACS Appl. Nano Mater..

[cit13] Drummond N. D., Zolyomi V., Fal’ko V. I. (2012). Phys. Rev. B: Condens. Matter Mater. Phys..

[cit14] Cahangirov S., Topsakal M., Aktürk E., Sąhin H., Ciraci S. (2009). Phys. Rev. Lett..

[cit15] Drissi L. B., Saidi E. H., Bousmina M., Fassi-Fehri O. (2012). DFT investigations of the hydrogenation effect on silicene/graphene hybrids. J. Phys.: Condens. Matter.

[cit16] Zanane F. Z., Sadki K., Drissi L. B., Saidi E. H., Bousmina M. (2022). Graphene multilayers nanoribbons with chirality from molecular dynamics. Mater. Sci. Eng., B.

[cit17] Bacon M., Bradley S. J., Nann T. (2014). Graphene quantum dots. Part. Part. Syst. Charact..

[cit18] Rajender G., Giri P. K. (2016). Formation mechanism of graphene quantum dots and their edge state conversion probed by photoluminescence and Raman spectroscopy. J. Mater. Chem. C.

[cit19] Zhu S., Song Y., Zhao X., Shao J., Zhang J., Yang B. (2015). The photoluminescence mechanism in carbon dots (graphene quantum dots, carbon nanodots, and polymer dots): current state and future perspective. Nano Res..

[cit20] Zeng M., Wang X., Yu Y. H., Zhang L., Shafi W., Huang X., Cheng Z. (2016). The synthesis of amphiphilic luminescent graphene quantum dot and its application in miniemulsion polymerization. J. Nanomater..

[cit21] Potasz P., Güçlü A. D., Voznyy O., Folk J. A., Hawrylak P. (2011). Electronic and magnetic properties of triangular graphene quantum rings. Phys. Rev. B: Condens. Matter Mater. Phys..

[cit22] Zhang Z. Z., Chang K., Peeters F. M. (2008). Tuning of energy levels and optical properties of graphene quantum dots. Phys. Rev. B: Condens. Matter Mater. Phys..

[cit23] Du Y., Guo S. (2016). Chemically doped fluorescent carbon and graphene quantum dots for bioimaging, sensor, catalytic and photoelectronic applications. Nanoscale.

[cit24] Li Y., Shu H., Wang S., Wang J. (2015). Electronic and optical properties of graphene quantum dots: the role of many-body effects. J. Phys. Chem. C.

[cit25] Yamijala S. S., Bandyopadhyay A., Pati S. K. (2014). Electronic properties of zigzag, armchair and their hybrid quantum dots of graphene and boron-nitride with and without substitution: a DFT study. Chem. Phys. Lett..

[cit26] Zhang R., Qi S., Jia J., Torre B., Zeng H., Wu H., Xu X. (2015). Size and refinement edge-shape effects of graphene quantum dots on UV-visible absorption. J. Alloys Compd..

[cit27] Li Y., Shu H., Niu X., Wang J. (2015). Electronic and optical properties of edge-functionalized graphene quantum dots and the underlying mechanism. J. Phys. Chem. C.

[cit28] Feng J., Dong H., Pang B., Shao F., Zhang C., Yu L., Dong L. (2018). Theoretical study on the optical and electronic properties of graphene quantum dots doped with heteroatoms. Phys. Chem. Chem. Phys..

[cit29] Sk M. A., Ananthanarayanan A., Huang L., Lim K. H., Chen P. (2014). Revealing the tunable photoluminescence properties of graphene quantum dots. J. Mater. Chem. C.

[cit30] Gu J., Zhang X., Pang A., Yang J. (2016). Facile synthesis and photoluminescence characteristics of blue-emitting nitrogen-doped graphene quantum dots. Nanotechnology.

[cit31] Li M., Wu W., Ren W., Cheng H. M., Tang N., Zhong W., Du Y. (2012). Synthesis and upconversion luminescence of N-doped graphene quantum dots. Appl. Phys. Lett..

[cit32] Niu X., Li Y., Shu H., Wang J. (2016). Revealing the underlying absorption and emission mechanism of nitrogen doped graphene quantum dots. Nanoscale.

[cit33] Gao S., Tang L., Xiang J., Ji R., Lai S. K., Yuan S., Lau S. P. (2017). Facile preparation of sulphur-doped graphene quantum dots for ultra-high performance ultraviolet photodetectors. New J. Chem..

[cit34] Li X., Lau S. P., Tang L., Ji R., Yang P. (2014). Sulphur doping: a facile approach to tune the electronic structure and optical properties of graphene quantum dots. Nanoscale.

[cit35] Li S., Li Y., Cao J., Zhu J., Fan L., Li X. (2014). Sulfur-doped graphene quantum dots as a novel fluorescent probe for highly selective and sensitive detection of Fe3+. Anal. Chem..

[cit36] Li Y., Li S., Wang Y., Wang J., Liu H., Liu X., Ma N. (2017). Electrochemical synthesis of phosphorus-doped graphene quantum dots for free radical scavenging. Phys. Chem. Chem. Phys..

[cit37] Wang G., Xu A., He P., Guo Q., Liu Z., Wang Z., Ding G. (2019). Green
preparation of lattice phosphorus doped graphene quantum dots with tunable emission wavelength for bio-imaging. Mater. Lett..

[cit38] Li Y., Zhao Y., Cheng H., Hu Y., Shi G., Dai L., Qu L. (2012). Nitrogen-doped graphene quantum dots with oxygen-rich functional groups. J. Am. Chem. Soc..

[cit39] Li Y., Hu Y., Zhao Y., Shi G., Deng L., Hou Y., Qu L. (2011). An electrochemical avenue to green-luminescent graphene quantum dots as potential electron-acceptors for photovoltaics. Adv. Mater..

[cit40] Feng J., Dong H., Pang B., Shao F., Zhang C., Yu L., Dong L. (2018). Theoretical study on the optical and electronic properties of graphene quantum dots doped with heteroatoms. Phys. Chem. Chem. Phys..

[cit41] Hedin L., Lundqvist B. I. (2064). Explicit local exchange-correlation potentials. J. Phys. C: Solid State Phys..

[cit42] Giannozzi P., Baroni S., Bonini N., Calandra M., Car R., Cavazzoni C., Ceresoli D., Chiarotti G. L., Cococcioni M., Dabo I., Dal Corso A. (2009). QUANTUM ESPRESSO: a modular and open-source software project for quantum simulations of materials. J. Phys.: Condens. Matter.

[cit43] Henkelman G., Arnaldsson A., Jónsson H. (2006). A fast and robust algorithm for Bader decomposition of charge density. Comput. Mater. Sci..

[cit44] Marini A., Hogan C., Grüning M., Yambo D. V. (2009). An *ab initio* tool for excited state calculations. Comput. Phys. Commun..

[cit45] Basak T., Chakraborty H., Shukla A. (2015). Theory of linear optical absorption in diamond-shaped graphene quantum dots. Phys. Rev. B: Condens. Matter Mater. Phys..

[cit46] Robertson J. M. (1948). Bond-length variations in aromatic systems. Acta Crystallogr..

[cit47] Havenith R. W., van Lenthe J. H., Dijkstra F., Jenneskens L. W. (2001). Aromaticity of Pyrene and Its Cyclopentafused Congeners Resonance and NICS Criteria. An *Ab Initio* Valence Bond Analysis in Terms of Kekulé Resonance Structures. J. Phys. Chem. A.

[cit48] Dias J. R. (1984). Isomer enumeration of nonradical strictly peri-condensed polycyclic aromatic hydrocarbons. Can. J. Chem..

[cit49] Parr R. G., Szentpály L. V., Liu S. (1999). Electrophilicity index. J. Am. Chem. Soc..

[cit50] Chen T., Zheng L., Yuan J., An Z., Chen R., Tao Y., Li H., Xie X., Huang W. (2015). Understanding the Control of Singlet-Triplet Splitting for Organic Exciton Manipulating: A Combined Theoretical and Experimental Approach. Sci. Rep..

[cit51] Ramadan F. Z., Ouarrad H., Drissi L. B. (2018). Tuning Optoelectronic Properties of the Graphene-Based Quantum Dots C16-x Six H10 Family. J. Phys. Chem. A.

[cit52] Ouarrad H., Ramadan F. Z., Drissi L. B. (2019). Size engineering optoelectronic features of C, Si and CSi hybrid diamond-shaped quantum dots. RSC Adv..

[cit53] Drissi L. B., Ouarrad H., Ramadan F. Z., Fritzsche W. (2020). Graphene and silicene quantum dots for nanomedical diagnostics. RSC Adv..

[cit54] Ouarrad H., Ramadan F. Z., Drissi L. B. (2020). Engineering silicon-carbide quantum dots for third generation photovoltaic cells. Opt. Express.

[cit55] Ouarrad H., Ramadan F. Z., Drissi L. B. (2022). Fluorescent quantum dots from two-dimensional nanomaterials for *in vitro* and *in vivo* bioimaging. Mater. Today: Proc..

[cit56] Li Y., Shu H., Niu X., Wang J. (2015). Electronic and optical properties of edge-functionalized graphene quantum dots and the underlying mechanism. J. Phys. Chem. C.

[cit57] Malloci G., Joblin C., Mulas G. (2007). On-line database of the spectral properties of polycyclic aromatic hydrocarbons. Chem. Phys..

[cit58] Koskinen P., Malola S., Häkkinen H. (2008). Self-passivating edge reconstructions of graphene. Phys. Rev. Lett..

[cit59] Girit Ç. Ö., Meyer J. C., Erni R., Rossell M. D., Kisielowski C., Yang L., Park C. H., Crommie M. F., Cohen M. L., Louie S. G., Zettl A. (2009). Graphene at the edge: stability and dynamics. Science.

[cit60] Senturk Dalgic S. (2016). Size dependent properties of hollow gold nanoparticles: a theoretical investigation. Acta Phys. Pol., A.

[cit61] Ekramipooya A., Valadi F. M., Farisabadi A., Gholami M. R. (2021). Effect of the heteroatom presence in different positions of the model asphaltene structure on the self-aggregation: MD and DFT study. J. Mol. Liq..

[cit62] Feng J., Guo Q., Song N., Liu H., Dong H., Chen Y., Yu L., Dong L. (2021). Density functional theory study on optical and electronic properties of co-doped graphene quantum dots based on different nitrogen doping patterns. Diamond Relat. Mater..

[cit63] Sarkar S., Sudolska M., Dubecky M., Reckmeier C. J., Rogach A. L., Zboril R., Otyepka M. (2016). Graphitic nitrogen doping in carbon dots causes red-shifted absorption. J. Phys. Chem. C.

[cit64] Feng J., Dong H., Pang B., Chen Y., Yu L., Dong L. (2019). Tuning the electronic and optical properties of graphene quantum dots by selective boroniction. J. Mater. Chem. C.

